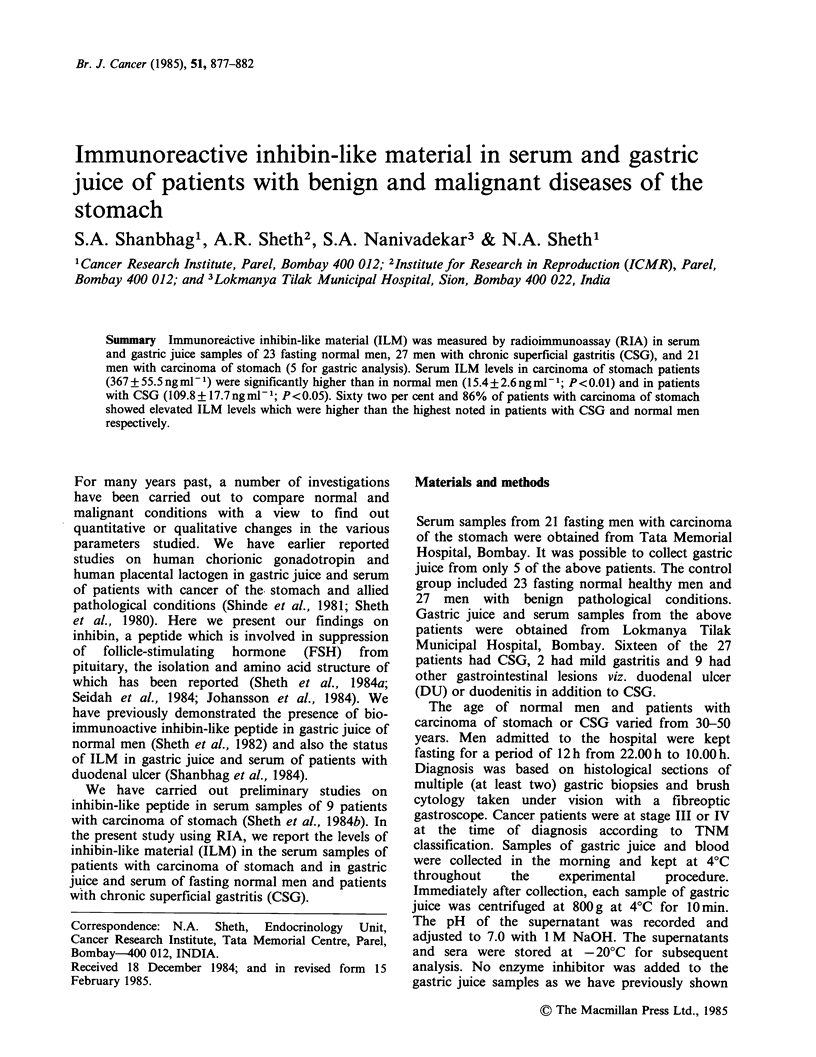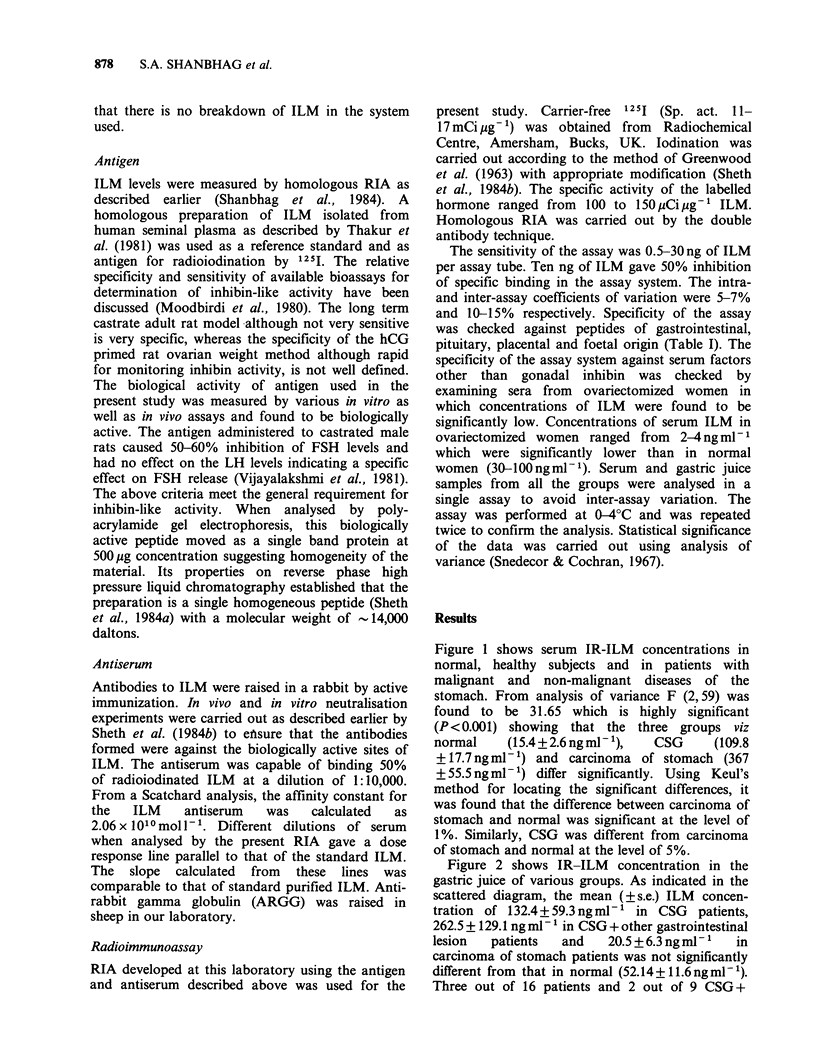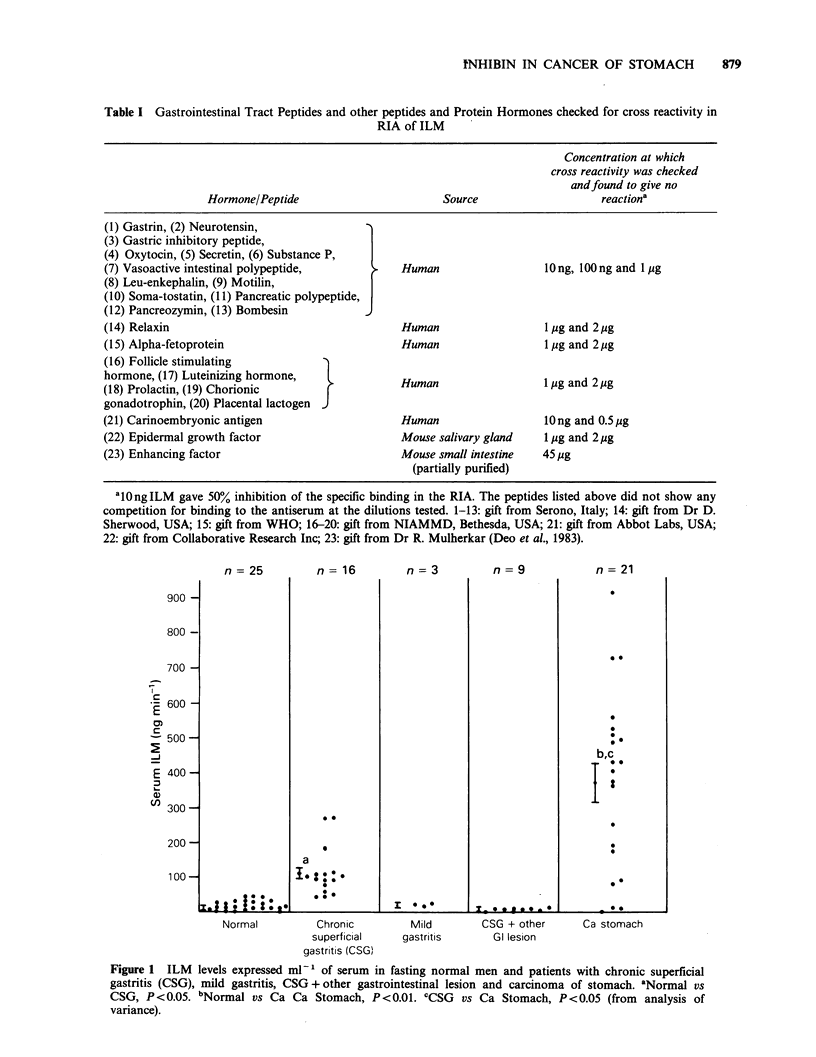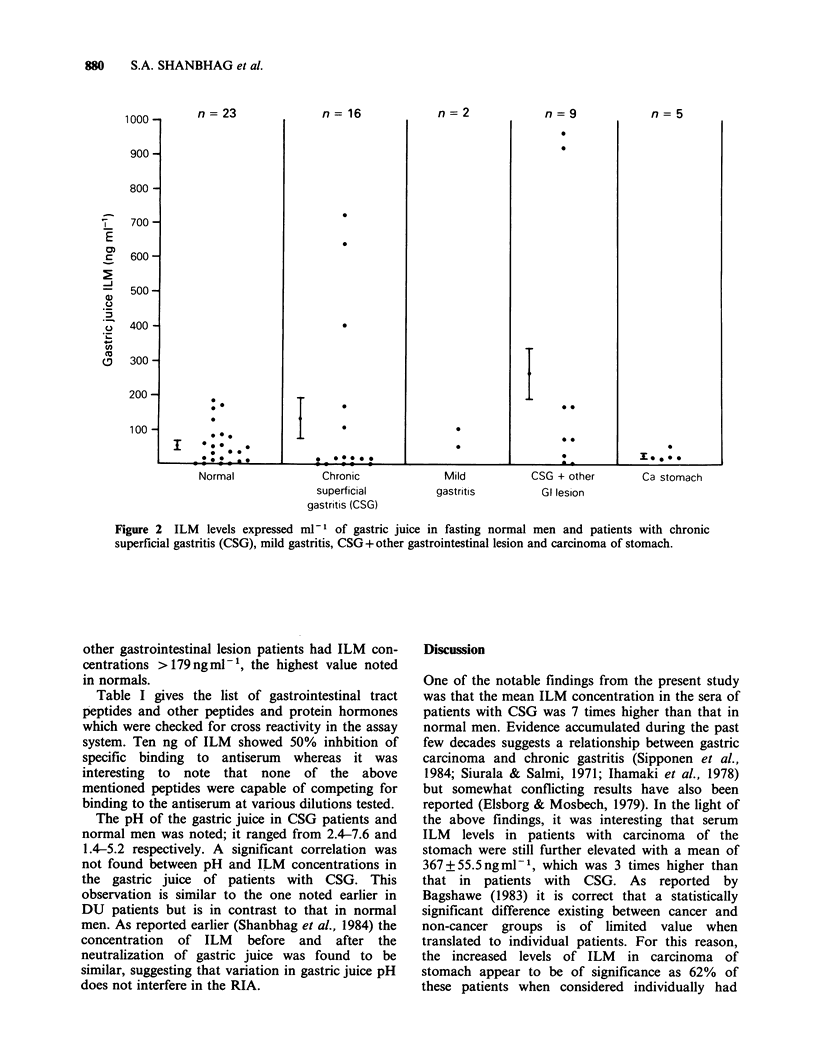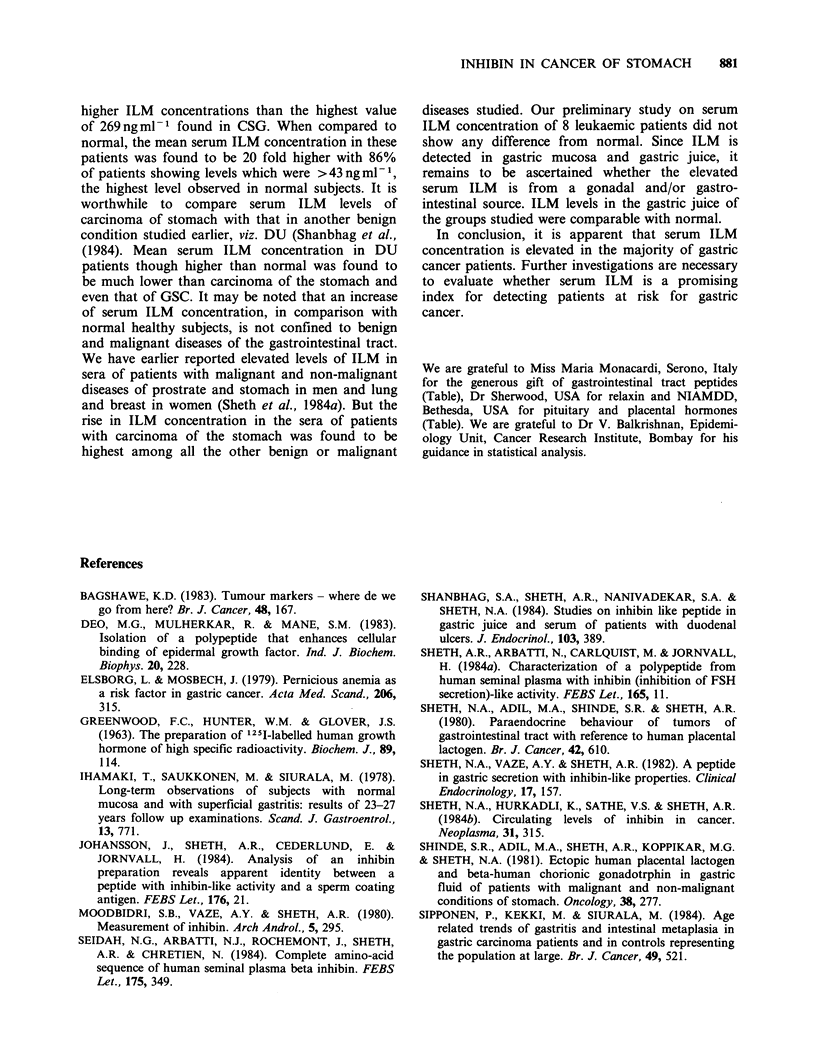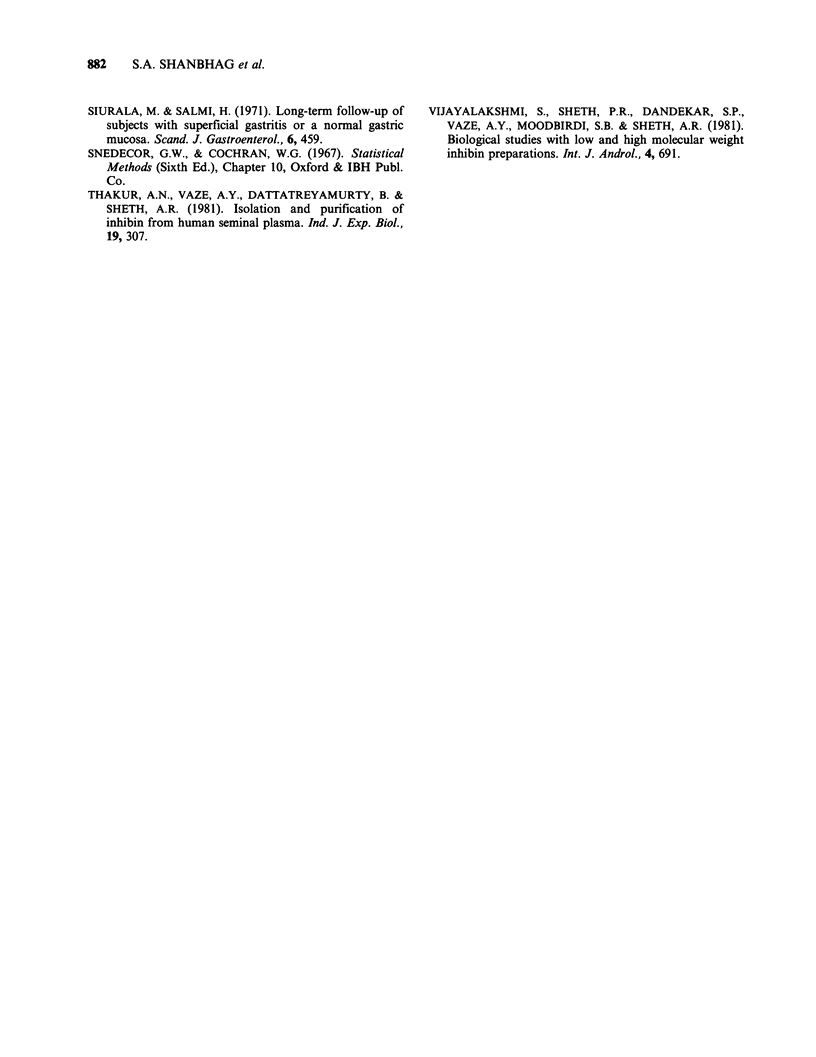# Immunoreactive inhibin-like material in serum and gastric juice of patients with benign and malignant diseases of the stomach.

**DOI:** 10.1038/bjc.1985.133

**Published:** 1985-06

**Authors:** S. A. Shanbhag, A. R. Sheth, S. A. Nanivadekar, N. A. Sheth

## Abstract

Immunoreactive inhibin-like material (ILM) was measured by radioimmunoassay (RIA) in serum and gastric juice samples of 23 fasting normal men, 27 men with chronic superficial gastritis (CSG), and 21 men with carcinoma of stomach (5 for gastric analysis). Serum ILM levels in carcinoma of stomach patients (367 +/- 55.5 ng ml-1) were significantly higher than in normal men (15.4 +/- 2.6 ng ml-1; P less than 0.01) and in patients with CSG (109.8 +/- 17.7 ng ml-1; P less than 0.05). Sixty two per cent and 86% of patients with carcinoma of stomach showed elevated ILM levels which were higher than the highest noted in patients with CSG and normal men respectively.


*Br. J. Cancer* (1985), **51**, 877–882

### Immunoreactive inhibin-like material in serum and gastric juice of patients with benign and malignant diseases of the stomach

S.A. Shanbhag[1], A.R. Sheth[2], S.A. Nanivadekar[3] & N.A. Sheth[1]

[1]*Cancer Research Institute, Parel, Bombay 400 012;* [2]*Institute for Research in Reproduction (ICMR), Parel, Bombay 400 012; and* [3]*Lokmanya Tilak Municipal Hospital, Sion, Bombay 400 022, India*

**Summary** Immunoreactive inhibin-like material (ILM) was measured by radioimmunoassay (RIA) in serum and gastric juice samples of 23 fasting normal men, 27 men with chronic superficial gastritis (CSG), and 21 men with carcinoma of stomach (5 for gastric analysis). Serum ILM levels in carcinoma of stomach patients ($367 \pm 55.5\,ng\,ml^{-1}$) were significantly higher than in normal men ($15.4 \pm 2.6\,ng\,ml^{-1}$; $P<0.01$) and in patients with CSG ($109.8 \pm 17.7\,ng\,ml^{-1}$; $P<0.05$). Sixty two per cent and 86% of patients with carcinoma of stomach showed elevated ILM levels which were higher than the highest noted in patients with CSG and normal men respectively.

For many years past, a number of investigations have been carried out to compare normal and malignant conditions with a view to find out quantitative or qualitative changes in the various parameters studied. We have earlier reported studies on human chorionic gonadotropin and human placental lactogen in gastric juice and serum of patients with cancer of the stomach and allied pathological conditions (Shinde *et al.*, 1981; Sheth *et al.*, 1980). Here we present our findings on inhibin, a peptide which is involved in suppression of follicle-stimulating hormone (FSH) from pituitary, the isolation and amino acid structure of which has been reported (Sheth *et al.*, 1984*a*; Seidah *et al.*, 1984; Johansson *et al.*, 1984). We have previously demonstrated the presence of bio-immunoactive inhibin-like peptide in gastric juice of normal men (Sheth *et al.*, 1982) and also the status of ILM in gastric juice and serum of patients with duodenal ulcer (Shanbhag *et al.*, 1984).

We have carried out preliminary studies on inhibin-like peptide in serum samples of 9 patients with carcinoma of stomach (Sheth *et al.*, 1984*b*). In the present study using RIA, we report the levels of inhibin-like material (ILM) in the serum samples of patients with carcinoma of stomach and in gastric juice and serum of fasting normal men and patients with chronic superficial gastritis (CSG).

Correspondence: N.A. Sheth, Endocrinology Unit, Cancer Research Institute, Tata Memorial Centre, Parel, Bombay—400 012, INDIA.

Received 18 December 1984; and in revised form 15 February 1985.

### Materials and methods

Serum samples from 21 fasting men with carcinoma of the stomach were obtained from Tata Memorial Hospital, Bombay. It was possible to collect gastric juice from only 5 of the above patients. The control group included 23 fasting normal healthy men and 27 men with benign pathological conditions. Gastric juice and serum samples from the above patients were obtained from Lokmanya Tilak Municipal Hospital, Bombay. Sixteen of the 27 patients had CSG, 2 had mild gastritis and 9 had other gastrointestinal lesions *viz.* duodenal ulcer (DU) or duodenitis in addition to CSG.

The age of normal men and patients with carcinoma of stomach or CSG varied from 30–50 years. Men admitted to the hospital were kept fasting for a period of 12 h from 22.00 h to 10.00 h. Diagnosis was based on histological sections of multiple (at least two) gastric biopsies and brush cytology taken under vision with a fibreoptic gastroscope. Cancer patients were at stage III or IV at the time of diagnosis according to TNM classification. Samples of gastric juice and blood were collected in the morning and kept at 4°C throughout the experimental procedure. Immediately after collection, each sample of gastric juice was centrifuged at 800 g at 4°C for 10 min. The pH of the supernatant was recorded and adjusted to 7.0 with 1 M NaOH. The supernatants and sera were stored at −20°C for subsequent analysis. No enzyme inhibitor was added to the gastric juice samples as we have previously shown

© The Macmillan Press Ltd., 1985

that there is no breakdown of ILM in the system used.

*Antigen*

ILM levels were measured by homologous RIA as described earlier (Shanbhag *et al.*, 1984). A homologous preparation of ILM isolated from human seminal plasma as described by Thakur *et al.* (1981) was used as a reference standard and as antigen for radioiodination by $^{125}$I. The relative specificity and sensitivity of available bioassays for determination of inhibin-like activity have been discussed (Moodbirdi *et al.*, 1980). The long term castrate adult rat model although not very sensitive is very specific, whereas the specificity of the hCG primed rat ovarian weight method although rapid for monitoring inhibin activity, is not well defined. The biological activity of antigen used in the present study was measured by various *in vitro* as well as *in vivo* assays and found to be biologically active. The antigen administered to castrated male rats caused 50–60% inhibition of FSH levels and had no effect on the LH levels indicating a specific effect on FSH release (Vijayalakshmi *et al.*, 1981). The above criteria meet the general requirement for inhibin-like activity. When analysed by polyacrylamide gel electrophoresis, this biologically active peptide moved as a single band protein at 500 $\mu$g concentration suggesting homogeneity of the material. Its properties on reverse phase high pressure liquid chromatography established that the preparation is a single homogeneous peptide (Sheth *et al.*, 1984*a*) with a molecular weight of $\sim$14,000 daltons.

*Antiserum*

Antibodies to ILM were raised in a rabbit by active immunization. *In vivo* and *in vitro* neutralisation experiments were carried out as described earlier by Sheth *et al.* (1984*b*) to ensure that the antibodies formed were against the biologically active sites of ILM. The antiserum was capable of binding 50% of radioiodinated ILM at a dilution of 1:10,000. From a Scatchard analysis, the affinity constant for the ILM antiserum was calculated as $2.06 \times 10^{10}$ mol l$^{-1}$. Different dilutions of serum when analysed by the present RIA gave a dose response line parallel to that of the standard ILM. The slope calculated from these lines was comparable to that of standard purified ILM. Anti-rabbit gamma globulin (ARGG) was raised in sheep in our laboratory.

*Radioimmunoassay*

RIA developed at this laboratory using the antigen and antiserum described above was used for the present study. Carrier-free $^{125}$I (Sp. act. 11–17 mCi $\mu$g$^{-1}$) was obtained from Radiochemical Centre, Amersham, Bucks, UK. Iodination was carried out according to the method of Greenwood *et al.* (1963) with appropriate modification (Sheth *et al.*, 1984*b*). The specific activity of the labelled hormone ranged from 100 to 150 $\mu$Ci $\mu$g$^{-1}$ ILM. Homologous RIA was carried out by the double antibody technique.

The sensitivity of the assay was 0.5–30 ng of ILM per assay tube. Ten ng of ILM gave 50% inhibition of specific binding in the assay system. The intra- and inter-assay coefficients of variation were 5–7% and 10–15% respectively. Specificity of the assay was checked against peptides of gastrointestinal, pituitary, placental and foetal origin (Table I). The specificity of the assay system against serum factors other than gonadal inhibin was checked by examining sera from ovariectomized women in which concentrations of ILM were found to be significantly low. Concentrations of serum ILM in ovariectomized women ranged from 2–4 ng ml$^{-1}$ which were significantly lower than in normal women (30–100 ng ml$^{-1}$). Serum and gastric juice samples from all the groups were analysed in a single assay to avoid inter-assay variation. The assay was performed at 0–4°C and was repeated twice to confirm the analysis. Statistical significance of the data was carried out using analysis of variance (Snedecor & Cochran, 1967).

### Results

Figure 1 shows serum IR-ILM concentrations in normal, healthy subjects and in patients with malignant and non-malignant diseases of the stomach. From analysis of variance F (2, 59) was found to be 31.65 which is highly significant ($P<0.001$) showing that the three groups *viz* normal ($15.4 \pm 2.6$ ng ml$^{-1}$), CSG ($109.8 \pm 17.7$ ng ml$^{-1}$) and carcinoma of stomach ($367 \pm 55.5$ ng ml$^{-1}$) differ significantly. Using Keul's method for locating the significant differences, it was found that the difference between carcinoma of stomach and normal was significant at the level of 1%. Similarly, CSG was different from carcinoma of stomach and normal at the level of 5%.

Figure 2 shows IR–ILM concentration in the gastric juice of various groups. As indicated in the scattered diagram, the mean ($\pm$s.e.) ILM concentration of $132.4 \pm 59.3$ ng ml$^{-1}$ in CSG patients, $262.5 \pm 129.1$ ng ml$^{-1}$ in CSG + other gastrointestinal lesion patients and $20.5 \pm 6.3$ ng ml$^{-1}$ in carcinoma of stomach patients was not significantly different from that in normal ($52.14 \pm 11.6$ ng ml$^{-1}$). Three out of 16 patients and 2 out of 9 CSG +

**Table I** Gastrointestinal Tract Peptides and other peptides and Protein Hormones checked for cross reactivity in RIA of ILM

| *Hormone/Peptide* | *Source* | *Concentration at which cross reactivity was checked and found to give no reaction*[a] |
|---|---|---|
| (1) Gastrin, (2) Neurotensin, (3) Gastric inhibitory peptide, (4) Oxytocin, (5) Secretin, (6) Substance P, (7) Vasoactive intestinal polypeptide, (8) Leu-enkephalin, (9) Motilin, (10) Soma-tostatin, (11) Pancreatic polypeptide, (12) Pancreozymin, (13) Bombesin | *Human* | 10 ng, 100 ng and 1 μg |
| (14) Relaxin | *Human* | 1 μg and 2 μg |
| (15) Alpha-fetoprotein | *Human* | 1 μg and 2 μg |
| (16) Follicle stimulating hormone, (17) Luteinizing hormone, (18) Prolactin, (19) Chorionic gonadotrophin, (20) Placental lactogen | *Human* | 1 μg and 2 μg |
| (21) Carinoembryonic antigen | *Human* | 10 ng and 0.5 μg |
| (22) Epidermal growth factor | *Mouse salivary gland* | 1 μg and 2 μg |
| (23) Enhancing factor | *Mouse small intestine* (partially purified) | 45 μg |

[a]10 ng ILM gave 50% inhibition of the specific binding in the RIA. The peptides listed above did not show any competition for binding to the antiserum at the dilutions tested. 1–13: gift from Serono, Italy; 14: gift from Dr D. Sherwood, USA; 15: gift from WHO; 16–20: gift from NIAMMD, Bethesda, USA; 21: gift from Abbot Labs, USA; 22: gift from Collaborative Research Inc; 23: gift from Dr R. Mulherkar (Deo *et al.*, 1983).

**Figure 1** ILM levels expressed $ml^{-1}$ of serum in fasting normal men and patients with chronic superficial gastritis (CSG), mild gastritis, CSG + other gastrointestinal lesion and carcinoma of stomach. [a]Normal *vs* CSG, $P<0.05$. [b]Normal *vs* Ca Ca Stomach, $P<0.01$. [c]CSG *vs* Ca Stomach, $P<0.05$ (from analysis of variance).

**Figure 2** ILM levels expressed $ml^{-1}$ of gastric juice in fasting normal men and patients with chronic superficial gastritis (CSG), mild gastritis, CSG + other gastrointestinal lesion and carcinoma of stomach.

other gastrointestinal lesion patients had ILM concentrations $>179\,ng\,ml^{-1}$, the highest value noted in normals.

Table I gives the list of gastrointestinal tract peptides and other peptides and protein hormones which were checked for cross reactivity in the assay system. Ten ng of ILM showed 50% inhbition of specific binding to antiserum whereas it was interesting to note that none of the above mentioned peptides were capable of competing for binding to the antiserum at various dilutions tested.

The pH of the gastric juice in CSG patients and normal men was noted; it ranged from 2.4–7.6 and 1.4–5.2 respectively. A significant correlation was not found between pH and ILM concentrations in the gastric juice of patients with CSG. This observation is similar to the one noted earlier in DU patients but is in contrast to that in normal men. As reported earlier (Shanbhag et al., 1984) the concentration of ILM before and after the neutralization of gastric juice was found to be similar, suggesting that variation in gastric juice pH does not interfere in the RIA.

### Discussion

One of the notable findings from the present study was that the mean ILM concentration in the sera of patients with CSG was 7 times higher than that in normal men. Evidence accumulated during the past few decades suggests a relationship between gastric carcinoma and chronic gastritis (Sipponen et al., 1984; Siurala & Salmi, 1971; Ihamaki et al., 1978) but somewhat conflicting results have also been reported (Elsborg & Mosbech, 1979). In the light of the above findings, it was interesting that serum ILM levels in patients with carcinoma of the stomach were still further elevated with a mean of $367 \pm 55.5\,ng\,ml^{-1}$, which was 3 times higher than that in patients with CSG. As reported by Bagshawe (1983) it is correct that a statistically significant difference existing between cancer and non-cancer groups is of limited value when translated to individual patients. For this reason, the increased levels of ILM in carcinoma of stomach appear to be of significance as 62% of these patients when considered individually had higher ILM concentrations than the highest value of 269 ng ml$^{-1}$ found in CSG. When compared to normal, the mean serum ILM concentration in these patients was found to be 20 fold higher with 86% of patients showing levels which were $>43$ ng ml$^{-1}$, the highest level observed in normal subjects. It is worthwhile to compare serum ILM levels of carcinoma of stomach with that in another benign condition studied earlier, *viz.* DU (Shanbhag *et al.*, (1984). Mean serum ILM concentration in DU patients though higher than normal was found to be much lower than carcinoma of the stomach and even that of GSC. It may be noted that an increase of serum ILM concentration, in comparison with normal healthy subjects, is not confined to benign and malignant diseases of the gastrointestinal tract. We have earlier reported elevated levels of ILM in sera of patients with malignant and non-malignant diseases of prostrate and stomach in men and lung and breast in women (Sheth *et al.*, 1984*a*). But the rise in ILM concentration in the sera of patients with carcinoma of the stomach was found to be highest among all the other benign or malignant diseases studied. Our preliminary study on serum ILM concentration of 8 leukaemic patients did not show any difference from normal. Since ILM is detected in gastric mucosa and gastric juice, it remains to be ascertained whether the elevated serum ILM is from a gonadal and/or gastrointestinal source. ILM levels in the gastric juice of the groups studied were comparable with normal.

In conclusion, it is apparent that serum ILM concentration is elevated in the majority of gastric cancer patients. Further investigations are necessary to evaluate whether serum ILM is a promising index for detecting patients at risk for gastric cancer.

We are grateful to Miss Maria Monacardi, Serono, Italy for the generous gift of gastrointestinal tract peptides (Table), Dr Sherwood, USA for relaxin and NIAMDD, Bethesda, USA for pituitary and placental hormones (Table). We are grateful to Dr V. Balkrishnan, Epidemiology Unit, Cancer Research Institute, Bombay for his guidance in statistical analysis.

### References

BAGSHAWE, K.D. (1983). Tumour markers – where de we go from here? *Br. J. Cancer*, **48**, 167.

DEO, M.G., MULHERKAR, R. & MANE, S.M. (1983). Isolation of a polypeptide that enhances cellular binding of epidermal growth factor. *Ind. J. Biochem. Biophys.* **20**, 228.

ELSBORG, L. & MOSBECH, J. (1979). Pernicious anemia as a risk factor in gastric cancer. *Acta Med. Scand.*, **206**, 315.

GREENWOOD, F.C., HUNTER, W.M. & GLOVER, J.S. (1963). The preparation of $^{125}$I-labelled human growth hormone of high specific radioactivity. *Biochem. J.*, **89**, 114.

IHAMAKI, T., SAUKKONEN, M. & SIURALA, M. (1978). Long-term observations of subjects with normal mucosa and with superficial gastritis: results of 23–27 years follow up examinations. *Scand. J. Gastroentrol.*, **13**, 771.

JOHANSSON, J., SHETH, A.R., CEDERLUND, E. & JORNVALL, H. (1984). Analysis of an inhibin preparation reveals apparent identity between a peptide with inhibin-like activity and a sperm coating antigen. *FEBS Let.*, **176**, 21.

MOODBIDRI, S.B., VAZE, A.Y. & SHETH, A.R. (1980). Measurement of inhibin. *Arch Androl.*, **5**, 295.

SEIDAH, N.G., ARBATTI, N.J., ROCHEMONT, J., SHETH, A.R. & CHRETIEN, N. (1984). Complete amino-acid sequence of human seminal plasma beta inhibin. *FEBS Let.*, **175**, 349.

SHANBHAG, S.A., SHETH, A.R., NANIVADEKAR, S.A. & SHETH, N.A. (1984). Studies on inhibin like peptide in gastric juice and serum of patients with duodenal ulcers. *J. Endocrinol.*, **103**, 389.

SHETH, A.R., ARBATTI, N., CARLQUIST, M. & JORNVALL, H. (1984*a*). Characterization of a polypeptide from human seminal plasma with inhibin (inhibition of FSH secretion)-like activity. *FEBS Let.*, **165**, 11.

SHETH, N.A., ADIL, M.A., SHINDE, S.R. & SHETH, A.R. (1980). Paraendocrine behaviour of tumors of gastrointestinal tract with reference to human placental lactogen. *Br. J. Cancer*, **42**, 610.

SHETH, N.A., VAZE, A.Y. & SHETH, A.R. (1982). A peptide in gastric secretion with inhibin-like properties. *Clinical Endocrinology*, **17**, 157.

SHETH, N.A., HURKADLI, K., SATHE, V.S. & SHETH, A.R. (1984*b*). Circulating levels of inhibin in cancer. *Neoplasma*, **31**, 315.

SHINDE, S.R., ADIL, M.A., SHETH, A.R., KOPPIKAR, M.G. & SHETH, N.A. (1981). Ectopic human placental lactogen and beta-human chorionic gonadotrphin in gastric fluid of patients with malignant and non-malignant conditions of stomach. *Oncology*, **38**, 277.

SIPPONEN, P., KEKKI, M. & SIURALA, M. (1984). Age related trends of gastritis and intestinal metaplasia in gastric carcinoma patients and in controls representing the population at large. *Br. J. Cancer*, **49**, 521.

SIURALA, M. & SALMI, H. (1971). Long-term follow-up of subjects with superficial gastritis or a normal gastric mucosa. *Scand. J. Gastroenterol.*, **6,** 459.

SNEDECOR, G.W., & COCHRAN, W.G. (1967). *Statistical Methods* (Sixth Ed.), Chapter 10, Oxford & IBH Publ. Co.

THAKUR, A.N., VAZE, A.Y., DATTATREYAMURTY, B. & SHETH, A.R. (1981). Isolation and purification of inhibin from human seminal plasma. *Ind. J. Exp. Biol.*, **19,** 307.

VIJAYALAKSHMI, S., SHETH, P.R., DANDEKAR, S.P., VAZE, A.Y., MOODBIRDI, S.B. & SHETH, A.R. (1981). Biological studies with low and high molecular weight inhibin preparations. *Int. J. Androl.*, **4,** 691.